# Repair for Congenital Macrostomia: Vermilion Square Flap Method

**DOI:** 10.1155/2014/480598

**Published:** 2014-05-29

**Authors:** Renuka Dhingra, Asheesh Dhingra, Dipti Munjal

**Affiliations:** ^1^Department of Paedodontics and Preventive Dentistry, Maharaja Ganga Singh Dental College & Research Centre, Sri Ganganagar, Rajasthan 335002, India; ^2^Jeewan Multispeciality Centre, 414/4 Jacobpura, Gurgaon 122001, India

## Abstract

Transverse facial clefts (macrostomia) are rare disorders that result when the embryonic mandibular and maxillary processes of the first branchial arch fail to fuse due to failure of mesodermal migration and merging to obliterate the embryonic grooves between the maxillary and mandibular processes to form the angle of the mouth at its normal anatomic position. Macrostomia may be seen alone or in association with other anomalies. It may be unilateral, extending along a line from the commissure to the tragus or bilateral. It is usually partial but rarely complete. Transverse facial clefts are more common in males and more common on the left side when unilateral. The goal of macrostomia reconstruction is to achieve functional, symmetrical, and accurate oral commissure with minimal scar. In this paper, we present a six-year-old girl with unilateral macrostomia with preauricular skin tags and malformation of pinna on ipsilateral side treated with vermillion-square flap method. The scar is placed at the upper lip. At two-month followup, the oral commissures are symmetric, the scars are inconspicuous, and the overall balance of facial contour and lip is excellent. We recommend this method for patients with mild to moderate macrostomia.

## 1. Introduction

Transverse facial or Tessier No. 7 cleft or congenital macrostomia is a rare congenital anomaly [1]. This developmental aberration results from failure of fusion of the maxillary and mandibular processes of the first branchial arch [2, 3]. This also explains the frequent association of transverse cleft with developmental anomalies of the first and second branchial arches [2]. Isolated transverse clefts are rare [3]. In most of the series in the literature transverse cleft accounts for less than 0.5% of all clefts. The affliction can vary from slight widening of the mouth to a cleft extending to the ear. But mostly these clefts are unilateral and do not extend beyond the anterior border of the masseter [4].

Transverse clefts develop either due to failure of the maxillary and mandibular processes to fuse or a disruption in the processes after fusing. Although the former is a more acceptable theory, Gorlin and others claim that post-merging tear is the cause [5, 6] and it is thought to be part of the manifestation of hemifacial microstomia, the second most common congenital craniofacial anomaly [2].

An incidence of about 1 in 60,000 births to 1 in 300,000 live births has been recorded [5]. Anderson's (1965) series of 3988 cases of facial clefts treated in Denmark over a period of 30 years gave a figure of 13 patients with macrostomia, an incidence of 0.3% of the total series [5]. Almost all of these patients had other associated anomalies making isolated cases a rarity.

At seven weeks of gestation the lips separate from the alveolar areas with the formations of a vestibule and the maxillary and mandibular swellings then merge laterally to form the cheeks. Incomplete union here results in macrostomia, which could be unilateral or bilateral. Other etiopathogeneses have been given including those of Mckenzie and Craig [4] who believe the defects of the first branchial arch arise from inadequate arterial blood supply occurring during a period of rapid and critical facial growth and development. It could vary from slight widening of the mouth to a cleft extending back to the ear; they are usually unilateral and do not extend beyond the anterior border of the masseter. [7] Many procedures have been developed for correction of this malformation [8–14]. Eguchi et al. [10] described a vermilion square flap technique in 8 patients with satisfactory results.

The goals of repair have been stated as follows: (1) reconstruction of the integrity and continuity of the oral sphincter by identifying and approximating the maxillary and mandibular portion of the orbicularis oris; (2) approximation of other facial musculature; (3) reconstruction of a natural looking and symmetric oral commissure; (4) reconstruction of symmetric lips; (5) closure of the defect in layers, that is, mucosa, muscle, subcutis, and skin; and (6) skin closure with minimal scarring [15, 16].

We report an excellent result of applying this technique on a 6-year-old girl with unilateral macrostomia.

## 2. Case Report

**Figure 1:**
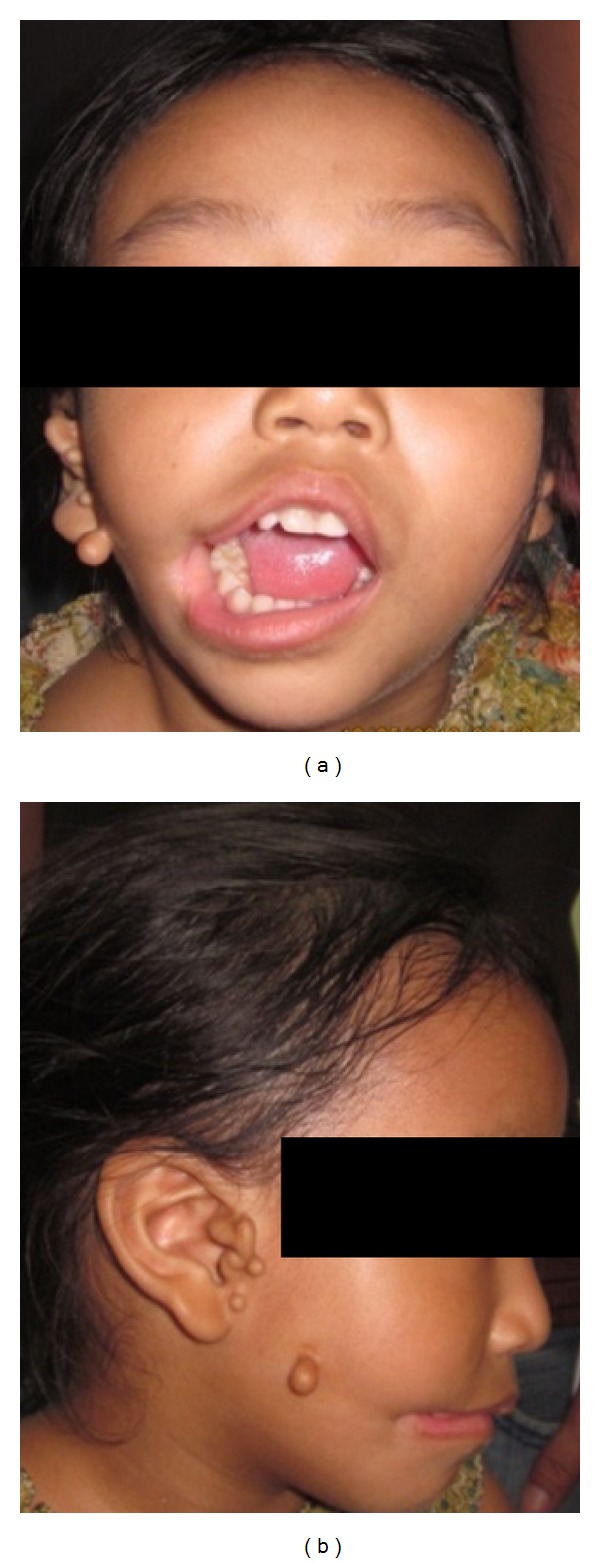
Preoperative photographs showing the frontal and lateral view of the patient.

A 6-year-old female patient visited the Department of Pedodontics and Preventive Dentistry with a chief complaint of wide mouth opening and preauricular skin tags since birth (Figures 1(a) and 1(b)). Antenatal and birth history was nonsignificant.

Patient was examined thoroughly to rule out other congenital anomalies and investigated for PAC. Patient was posted for surgery under general anesthesia with nasal intubation with north pole tube.

**Figure 2:**
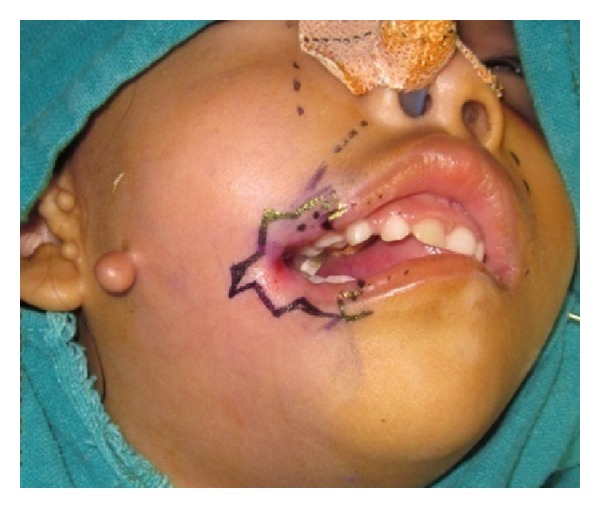
Photograph showing marking for the vermilion flap.

Surgical procedure: by careful inspection of the muscle and vermilion, the site of the new commissure was marked at the junction of the normal and abnormal vermilion where the muscle bundles end and the white line thins in line with the pupil with patient in horizontal gaze. A 3 mm × 3 mm mucocutaneous vermilion flap, which involves the vermilion border with its pedicle on the lower lip, was designed at the normal vermilion (Figure 2).

**Figure 3:**
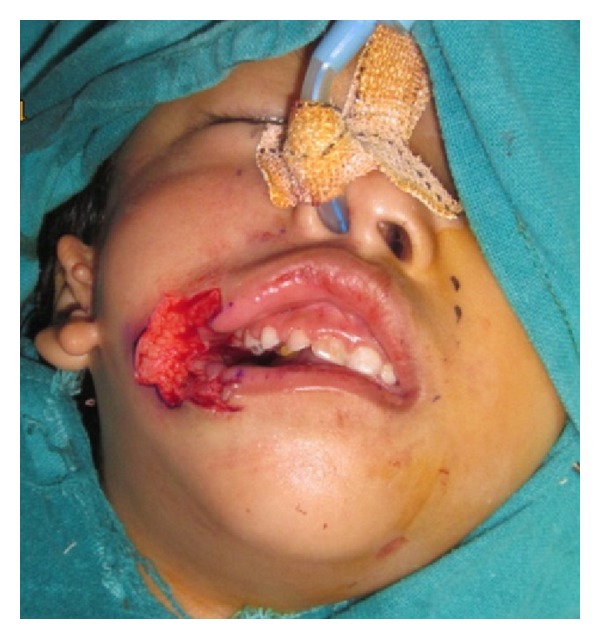
Photograph after flap elevation.

A lazy W-plasty was designed for closure of the skin of the cleft cheek. Incisions were made after infiltration of adrenaline solution in concentration 1 in 200,000 and the excess tissue is excised. The vermilion square flap on the lower lip was elevated. The orbicularis oris muscle of upper and lower lips, which is found in parallel fashion, was identified and exposed (Figure 3); buccal mucosa was trimmed and closed with vicryl 3/0 in a straight-line fashion up to the commissure.

**Figure 4:**
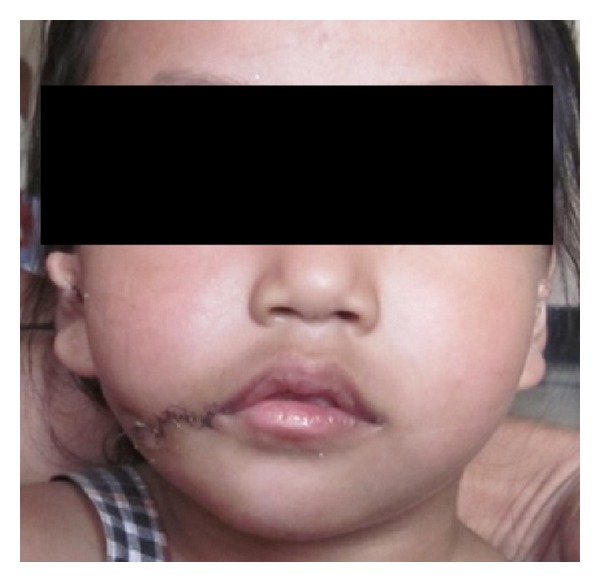
Postoperative photograph 7 days after surgery.

At the mouth angle, in order to reconstruct the continuity of the orbicularis oris muscle, the muscular bundle at the upper lip was placed over the inferior muscular bundle as suggested by Eguchi and others and they were sutured with pds 3/0 suture. The remaining muscle bundles at the cleft cheek were sutured into a side-to-side fashion. The vermilion square flap was transposed to the upper lip. The skin was then closed with ethilon 5/0 suture. At seventh postoperative day (Figure 4) stitches were removed.

**Figure 5:**
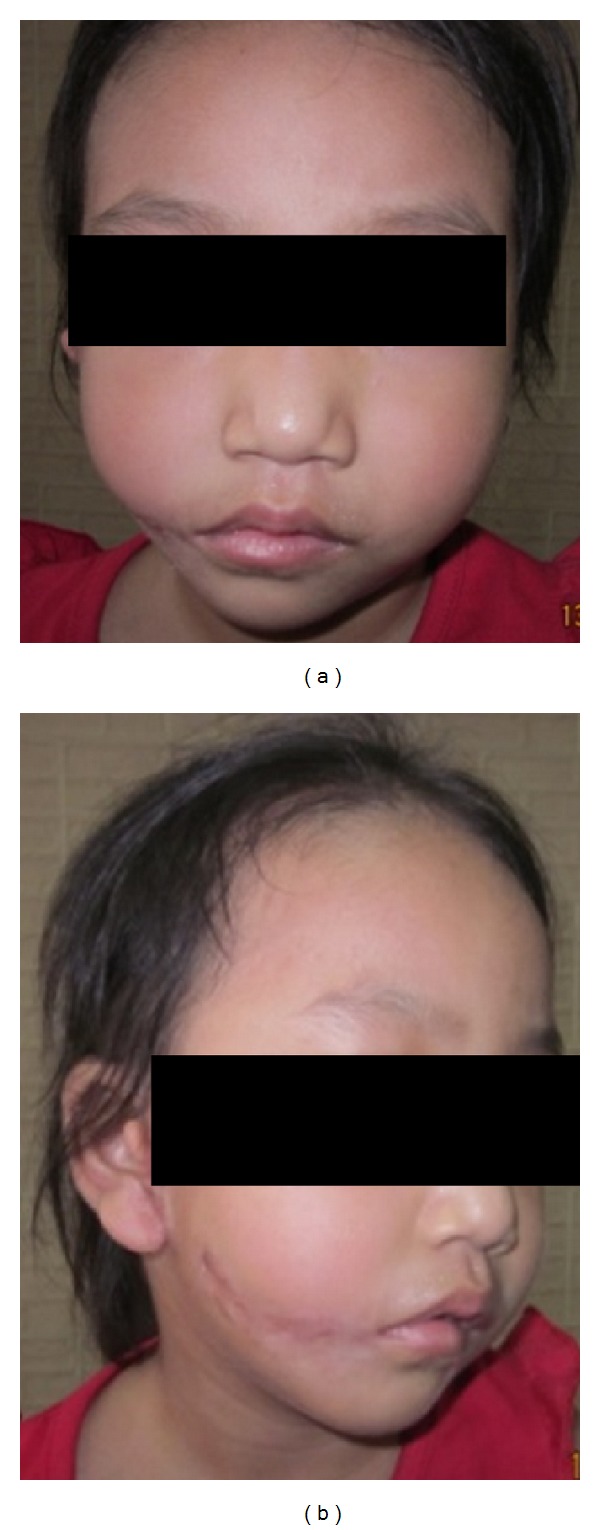
Postoperative frontal and lateral view.

Patient was followed up weekly for the first month and thereafter monthly for 2 months (Figures 5(a) and 5(b)).

## 3. Discussion 

Macrostomia is a relatively rare congenital craniofacial defect. It is more commonly unilateral than bilateral. It is not surprising that the condition is usually associated with other defects because of the many facial structures developing simultaneously. Almost always present is malformation of the mandible and/or the ear [5].

The factors that could be responsible for the development of macrostomia are genetic and environmental; in individual cases, however, as in the case presented here, it is often impossible to identify a specific aetiological factor. In this case there was no history of medication, use of traditional medications, illnesses or nutritional deficiencies in pregnancy, and no evidence of attempted abortion was established [17].

It is hoped that with public campaign and enlightenment activities, societal attitudes to children with congenital deformities will change. It is also hoped that the authorities will enact and enforce laws to ensure the rights of such babies, especially right to care and life [18].

Anatomically and functionally, the orbicularis oris muscle can be divided into two distinctive layers, superficial and deep [19]. The deep layer of orbicularis oris muscle consists of fibers that arise from other facial muscles. The upper muscle fibers of the deep layer decussate into the lower lip, and the lower muscle fibers decussate into the upper lip. The deep component has a sphincteric function. The superficial portion associates with the maxilla and septum above and the mandible below. It is believed that the superficial component functions during facial expression and provides the precise movements of the lips necessary for complex speech production.

The cheek deformity of macrostomia includes defects of three layers: skin, muscle, and mucosa. An interruption of orbicularis oris muscle results in compromised sphincteric function and facial expression. The aim of surgical correction is reconstruction of a functional oral musculature, accurate positioning without contractile scar of the oral commissure, and cheek skin closure with a minimal visible scar.

Many procedures are described like cutaneous Z-plasty [20], straight line closure [21] and two triangular flaps, advancement of oral commissure using a composite flap [22], W-plasty, and commissuroplasty by vermilion square flap which are common methods.

Initial workers used Z-plasty to close transverse facial cleft [23, 24] but later on it was noticed that the Z-plasty left a more visible scar. This drawback of Z-plasty led to development of straight line closure [25].

Kawai et al. described a technique to correct macrostomia with a simple straight line incision and incorporation of a small triangular flap to achieve proper positioning of the commissure with minimal visible scar [12].

The technique of two triangular flaps allows achieving all therapeutic goals, formation of symmetric lips and commissures of the mouth, reconstruction of the orbicularis muscle of mouth to restore labial function, and reconstruction of the commissure of the mouth with a natural looking contour. The advantage of this technique is that the position of the commissure of the mouth can be adjusted intraoperatively according to the extent of macrostomia.

All these methods emphasize the importance of restoration of the integrity of cheek and lip muscle. However, these methods of reconstruction have their scars located at the angle. Because the corner of the normal mouth is not a corner but rather a smooth and continuous segment of the vermilion [26], it is difficult to achieve a natural contour of the corner of the mouth when the scar is located at the angle.

Kaplan [27] reported commissuroplasty with a square flap from the upper lip which leaves a scar on the lower lip, not at the angle. Scar on the lower lip becomes more conspicuous over time because of the tension that is created when the mouth is opened.

The point of new commissure must be predetermined with accuracy and preoperative markings of the normal landmarks are very important. Eguchi et al. [10] described a vermilion square flap surgical technique that combines a lower lip mucocutaneous vermilion border flap with a lazy W-plasty to ensure a natural commissure and cheek skin closure. We used Eguchi's vermilion square flap technique to correct unilateral macrostomia.

By using the vermilion square flap pedicled on the lower lip, a scar at the commissural point which may lead to deformity of the oral commissure can be avoided. This technique in our experience is easy and the result is excellent. At 2-month followup, the oral commissures are symmetric. Natural contour of the oral commissure is obtained both at rest and during movement.

To conclude this method is a natural evolution from the earlier methods where the square flap pedicled on lower lip provides a natural shape to commissure avoiding sharp unnatural commissure margins and places scar such that the forces of muscle contraction and scar contracture balance out, thus maintaining the results to be more consistent on long-term followup.
